# Supplementary material for: Chromothripsis during telomere crisis is independent of NHEJ, and consistent with a replicative origin
Source: Genome Res. 2019 May;29(5):737–49. doi: 10.1101/gr.240705.118 (PMC6499312; doi:10.1101/gr.240705.118)
Supplement: Supplemental Material [file supp_gr.240705.118_Supplemental_file_1.zip › contigs/annotated_contigs/DB92/contig.2.DB92_length_614_mean_cov_6.69706840391.docx]

**DB92_length_614_mean_cov_6.69706840391**

```
CATTGAATAAGTAATTATGAGTGATGATAAATGTCACAGAAGGGTAATTCAGAGTGCTACAGAAGTGTATGAGGATGGGACCGAACATG
 >chr2:136497541-136497923 - E=3e-218
GGCTAGTGGAGTCAGAAAGGGCCTTCATGAGGAAATGACGTGTCGGGGAACTCTAGGTAGGTGAAGGAGAAGGCTATTCCACATGAGGC

TCTGAAGCAAGCAAACACAGTGCACTTGCAGAACTAAAAAATGCCCAGTGTGGCTATAACATAGAAAGAGGAGTGTGGTACAAGTGAGG

CTGGAAAGCTAGGCAAGGATCAGATGATACAGAGTCTTGAAAATCATGTTAAAAATTTTAAGACATTTTTCTAAGGATTACAGGGAGCC

ATTGAAAAATTTTAAACAGAAGAGTT|T|AATATGTTATATTTAATAAATTATATTAATATGGTTTTTAAGAAATGTTTGCTAAAAGTC
                            >chr2:136679490-136679721 - E=3e-118
AAGCACAGGCACATAATCATGTGGCAAATTCTTTTTTGAAGCTAATTGTAAGTGAAAAGCCAGCATTACTTTATTTAACACTTACCAAG

TTATTTTATATATGTTTTTTGATACCTTAATAAAAATTAAATACTTTCTAATAGATAAACTGGCAAGATCATTAAGGGTCTT
```
